# Improving retention and performance in civil society in Uganda

**DOI:** 10.1186/1478-4491-6-11

**Published:** 2008-06-20

**Authors:** Mary L O'Neil, Michael Paydos

**Affiliations:** 1 Center for Leadership and Management, Management Sciences for Health, Cambridge, MA, USA; 2 Leadership, Management, and Sustainability Program, Management Sciences for Health, Cambridge, MA, USA

## Abstract

This article is the second article in the Human Resources for Health journal's first quarterly feature. The series of seven articles has been contributed by Management Sciences for Health (MSH) under the theme of leadership and management in public health and will be published article-by-article over the next few weeks. The journal invited Dr Manuel M. Dayrit, Director of the WHO Department of Human Resources for Health and former Minister of Health for the Philippines to launch the feature with an opening editorial to be found in the journal's blog.

This article – number two in the series – describes the experience of the Family Life Education Programme (FLEP), a reproductive health program that provides community-based health services through 40 clinics in five districts of Uganda, in improving retention and performance by using the Management Sciences for Health (MSH) Human Resource Management Rapid Assessment Tool.

A few years ago, the FLEP of Busoga Diocese began to see an increase in staff turnover and a decrease in overall organizational performance. The workplace climate was poor and people stopped coming for services even though there were few other choices in the area. An external assessment found the quality of the health care services provided was deficient.

An action plan to improve their human resource management (HRM) system was developed and implemented. To assess the strengths and weaknesses of their system and to develop an action plan, they used the Rapid Assessment Tool. The tool guides users through a process of prioritizing and action planning after the assessment is done.

By implementing the various recommended changes, FLEP established an improved, responsive HRM system. Increased employee satisfaction led to less staff turnover, better performance, and increased utilization of health services. These benefits were achieved by cost-effective measures focused on professionalizing the organization's approach to HRM.

## Introduction

The Family Life Education Program (FLEP) of the Busoga Diocese of Uganda, a multi-service reproductive health agency that operates 40 rural clinics in five districts of Uganda was supported in part by the DISH II project, a project funded by the US Agency for International Development (USAID) [1]. FLEP provides community-based health services, including family planning, immunization, maternal health, nutrition, and HIV & AIDS counseling. At the end of the DISH II project, the services of FLEP were continued by the Planning and Development Department of Busoga Diocese.

When the program began to see an increase in staff turnover and a decrease in overall organizational performance, MSH was asked to help. The workplace climate was poor and people had stopped coming for services. FLEP's leaders decided it was time to examine their HRM system and practices.

In August 2001, the senior managers at FLEP used MSH's HRM Assessment Tool to examine the functioning of their HRM system. This tool provides users with a rapid way to identify the strengths and weaknesses of their HRM system and develop an action plan for improvement. The exercise, including the action plan, can be completed in one day.

The instrument consists of a matrix of 23 HRM components that fall into six broad areas of HRM:

### 1. HRM capacity

▪ HRM budget and staff

### 2. HR planning

▪ organizational mission and goals

▪ HR planning

### 3. Personnel policy and practice

▪ job classification system

▪ compensation and benefits system

▪ recruitment, hiring, transfer, and promotion

▪ orientation program

▪ policy manual

▪ discipline, termination, and grievance procedures

▪ relationships with unions

▪ labor law compliance

### 4. HRM Data

▪ employee data

▪ computerization of data

▪ personnel files

### 5. Performance Management

▪ job descriptions

▪ staff supervision

▪ work planning and performance review

### 6. Training

▪ staff training

▪ management and leadership development

▪ links to external pre-service training

The tool also describes four stages of development for each component and provides blank spaces for users to write a brief statement, or indicator, to show how the organization fits into a particular stage of development.

## Discussion

After the assessment, FLEP's management committee reviewed the results and determined the priorities for action. Their priorities were to:

▪ revise and update the personnel policy manual;

▪ complete personnel files and make them open to staff;

▪ update job descriptions;

▪ develop a new process for performance appraisal and strengthen supervision;

▪ improve communications among the 40 far-flung clinics.

Longer-range priorities were likewise planned for staff training, strengthening of management and leadership at all levels of the organization, and annual reviews of salary policy. A survey measuring employee satisfaction was carried out in September 2001 to identify other areas for intervention. In addition, FLEP managers worked with MSH to develop a monitoring and evaluation plan using indicators that would track HR management and performance components.

MSH staff and the FLEP Human Resource Administrator met with two representatives of the FLEP board of directors and briefed them on the HRM assessment, priorities identified, proposed actions, and indicators to measure performance improvement. The board members agreed that the need to improve FLEP's HRM was urgent, and they fully supported the proposed HR plan and new management approach.

### The Achievements of the Program

With MSH technical assistance, FLEP established a responsive HRM system. FLEP revised and updated its personnel policy and procedures, and produced and distributed a new personnel manual to management and supervisory staff at the clinics. Personnel files were completed and job descriptions were updated. A senior management team was installed at headquarters. Operations were streamlined by reducing the number of zonal coordinators from eight to four and Volunteer Health Worker supervisors from seventeen to eight. Poor-performing staff were dismissed and the remaining staff were given fixed contracts until the end of the project, which gave them an increased sense of security. A performance appraisal process was instituted, and supervisors were trained in basic supervision skills and the use of appraisal forms and a supervisory checklist.

These measures did not require additional funding or resources. Rather they reflected the commitment of the leadership team to support health staff by increasing equity, accountability, and opportunity in the workplace.

**Figure 1:**
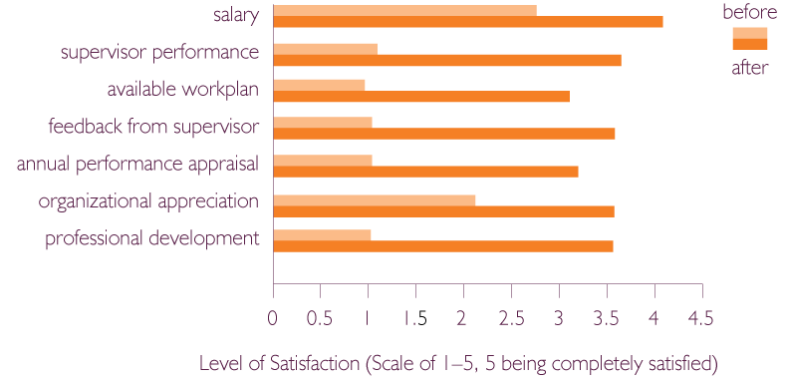
Improved employee satisfaction due to better human resource management.

**Figure 2:**
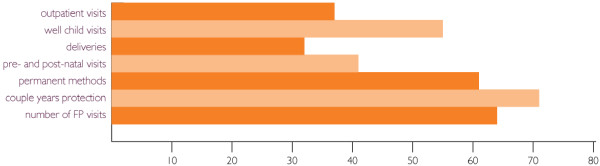
Increased utilization of services.

When the staff satisfaction and organizational survey was conducted again, in June 2002, it revealed significant improvements in ten (83%) of the twelve indicators, including employee satisfaction and commitment (see Figure 1). Two indicators of staff satisfaction did not increase: staff benefits and accuracy of job description, items that required more resources to address. In just one year's time, the functioning of management systems and delivery of health services improved significantly (see Figure 2). While FLEP's transformation may not be typical of that of all nongovernmental organizations, MSH's experience offers important insights – not only into the linkages between strong internal leadership, strengthened management systems, better work climate, and improved health services, but also in the ability of an organization to persevere through difficult times.

## Conclusion

The factors that contributed to this program's success were:

▪ a visionary leader who involved teams at all levels;

▪ establishing priorities based on assessment and root cause analysis;

▪ creating a climate of support for managers who were formerly isolated;

▪ establishing standards of performance and rewarding people for meeting or exceeding them;

▪ linking change to HRM systems.

The individuals leading this and similar transformations are not extraordinary. They are often doctors who have spent their careers working to improve health in their countries. What makes them effective in getting results is their commitment to addressing the human resource crisis and move from vision to action. In the process, they work to enable others to face challenges and achieve results, understanding that implementing change in human resources requires new ways of working.

## Competing interests

The authors declare that they have no competing interests.
